# A Portrait of the Sialyl Glycan Receptor Specificity of the H10 Influenza Virus Hemagglutinin—A Picture of an Avian Virus on the Verge of Becoming a Pandemic?

**DOI:** 10.3390/vaccines5040051

**Published:** 2017-12-13

**Authors:** Elena K. Schneider, Jian Li, Tony Velkov

**Affiliations:** 1 Drug Delivery, Disposition and Dynamics, Monash Institute of Pharmaceutical Sciences, Monash University, 381 Royal Parade, Parkville, VIC 3052, Australia; elena.schneider@unimelb.edu.au; 2 Department of Pharmacology & Therapeutics, School of Biomedical Sciences, Faculty of Medicine, Dentistry and Health Sciences, The University of Melbourne, Parkville, VIC 3010, Australia; 3 Monash Biomedicine Discovery Institute, Department of Microbiology, Monash University, Clayton, VIC 3800, Australia; jian.li@monash.edu

**Keywords:** influenza, H10, glycan specificity, hemagglutinin

## Abstract

Pandemic influenza is a constant global threat to human health. In particular, the pandemic potential of novel avian influenza viruses such as the H10N7 and H10N8 avian strains, which recently managed to cross the species barrier from birds to humans, are always of great concern as we are unlikely to have any prior immunity. Human and avian isolates of H10 influenza display the ability to rapidly adapt to replication in mammalian hosts. Fortunately, so far there is no evidence of efficient human-to-human transmission of any avian influenza virus. This review examines all of the available clinical and biological data for H10 influenza viruses with an emphasis on hemagglutinin as it is a major viral antigen that determines host range and immunity. The available glycan binding data on the influenza H10 hemagglutinin are discussed in a structure-recognition perspective. Importantly, this review raises the question of whether the emerging novel avian H10 influenza viruses truly represents a threat to global health that warrants close monitoring.

## 1. Overview of Influenza Viruses

The influenza viruses belong to the family of orthomyxoviride [1,2]. The single-stranded negative sense RNA genome consists of eight segments that encode 11 viral proteins [1,2]. There are two surface glycoproteins, hemagglutinin (HA) and neuraminidase (NA), and nine internal proteins [1,2]. In the known strains of influenza A viruses there are 18 HA sub-types [(H1-H18) 16 in birds and two in bats] and 11 NA subtypes (nine in birds and two in bats) [1,2,3,4]. Based on molecular phylogenies, HAs are divided into two groups; Group 1: H1, H2, H5, H6, H8, H9, H11, H12, H13, H16, H17 and H18; Group 2: H3, H4, H7, H10, H14 and H15 [5,6]. 

Influenza viral infections are responsible for recurrent annual epidemics and on the order of 250,000 to 500,000 deaths globally [7]. Recurring seasonal epidemics occur because the influenza virus is very adept at accumulating point mutations in the HA and NA genes during circulation, a process known as “antigenic drift”, which allows the virus to escape host immunity. Seasonal infection with influenza viruses allows our immune system to develop antibodies against the HA and NA, thereby providing the human population with a level of protective immunity against similar strains. The situation is much more serious with the introduction of viruses with novel HA or NA subtypes that have not previously been circulating in the human population. These viruses pose a serious pandemic threat as our immune system is totally naive and human infection is often associated with high levels of morbidity. The H1, H2 and H3 influenza subtypes have circulated in the human population for over a century, so the general population has some prior immunity against seasonal strains from these sub-types. On the other hand, avian influenza viruses such as the H5, H6, H7, H9 and H10 sub-types have novel HAs on their surface that will be largely foreign to the immune system of most of the human population. Therefore, vulnerability to novel avian influenza strains such as H10 could be global [8,9]. This review covers the available clinical and molecular level HA knowledge base on recent human infections with H10 influenza viruses, which represent an emerging threat to human health. 

## 2. Wild Bird Populations—A Reservoir for the H10 Influenza Virus 

**Figure 1:**
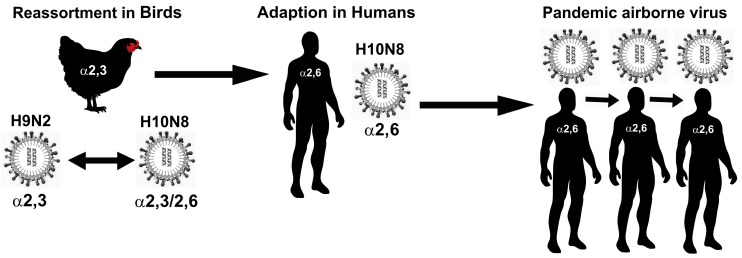
Zoonotic transmission of influenza H10 from birds to humans: a potential pandemic pathway.

Phylogenetic analysis of the H10 HA sequences available in the NCBI Influenza Virus Resource Database revealed that the H10 viruses can be divided into North American and Eurasian lineages [10]. A phylogenetic trace of the history of H10N8 suggests that the virus spilled over from wild birds to poultry in south China [10]. Wild wetland birds are natural reservoirs of avian influenza viruses [11,12]. Worryingly, outside of China the avian influenza H10 viruses (largely H10N7), appear to be endemic in wild bird populations and have been detected in Italy, the United States, South Africa, Canada, South Korea, Sweden and Japan since 1965 [13,14,15,16,17]. The low pathogenicity of the H10 viruses in birds makes them difficult to detect, allowing the virus to persist and spread; meaning wild birds and poultry can act as large reservoirs for the virus [18]. This situation increases the potential for the virus to develop mutations and undergo reassortment, endowing it with high pathogenicity and improved transmissibility to humans (Figure 1) [19,20]. 

Southern China has long been considered as an epicentre for emergence of pandemic influenza viruses in the world [21,22,23,24]. A Dongting Lake H10N8 isolate, A/environment/Dongting Lake/Hunan/3-9/07, showed low pathogenicity in chickens; however, the virus adapted quickly and replicated efficiently in mouse lung [25]. What is even more worrying is that the virus’s pathogenicity increased rapidly during lung adaptation, becoming lethal to mice after only two passages. Sequence analysis revealed that during adaption the strain underwent multiple amino acid substitutions in a number of genes. This is not surprising as it is becoming increasingly apparent that the host specificity and pathogenicity of influenza A viruses is determined by multiple genes [2,26]. The fact that this H10N8 strain replicated efficiently in the mouse lung without prior adaptation is worrying. A report from the Taubenberger laboratory at the NIAID showed that avian influenza HA subtypes H1, H6, H7, H10 and H15 contain inherent mammalian virulence factors similar to those of the pandemic 1918 virus [8]. Therefore, the potential exists that avian viruses expressing one of these HA subtypes could cause enhanced disease in humans. 

Wild birds are not the only possible reservoir for the H10 influenza virus; notably, avian H10N8 has been reported to infect feral dogs in live poultry markets in Guangdong Province, China and a H10N7 virus was involved in the mass mortality of harbor seals in Denmark, Sweden, Germany and the Netherlands [27,28].

## 3. Avian H10 Influenza—A Historic Timeline of Human Infection

The timeline of human cases of avian influenza virus infection is interesting; there were human cases of avian influenza infection reported as early as 1959 [29,30]. In 1997 the avian H5N1 caused an epidemic in Hong Kong [31]. The H5N1 virus then re-emerged in 2003 with a morbidity rate of 60% [32]. Periodic avian H9N2 occurred from 1998 [33]. In February 2013 a H7N9 avian influenza virus emerged in China [34,35]. The first confirmed human infections of avian H6N1 were reported in 2013 [36]. We are now faced with novel H10 avian influenza strains that have recently managed to cross the species barrier into humans. The following discussions provide an overview of the timeline of human H10 virus infections.

In 2004 H10N7 was detected in two Egyptian infants [37]. Coincidentally, the father of one of the infants is a poultry merchant who frequently travelled to a nearby town, Damietta, where five cases of H10N7 were isolated from hunted wild ducks in a live bird market between the 18th and 22nd of April 2004.

In 2012, two Australian poultry abattoir workers tested positive for H10N7 after they processed chickens from a farm associated with a 2010 H10N7 poultry outbreak [38]. The patients only exhibited mild clinical symptoms (conjunctivitis and minor upper respiratory tract symptoms) and made a full recovery. Genomic analysis has revealed that H10N7 viruses endemic in Australian poultry are similar to the strains circulating in aquatic birds since 2009; their initial transmission from migratory bird species into Australia likely occurred around 2007–2008 [39]. This is worrying as it shows that the virus rapidly spilled into poultry.

A 2014 report in the *Lancet* described a fatal H10N8 infection in a 73-year-old woman in China [40]. This was the first human death arising from infection with a H10 avian influenza virus and occurred in Jiangxi province. The elderly woman had a number of chronic underlying illnesses and was immune-compromised; therefore, it is not clear how deadly the infection would be in a healthy individual. The woman apparently contracted the infection after visiting a live-poultry market. She was admitted to a local hospital on November the 30th, where she died from severe pneumonia and multiple organ failure nine days after the initial onset of symptoms [40,41]. The virus, A/Jiangxi-Donghu/346/2013 (designated as JX346 from hereon in), was characterised by sequencing of tracheal aspirate viral samples. 

The JX346 virus harbours mutations at positions 99 and 368 and 627 in its PB2 gene, which are coincident with enhanced replication in ferrets and mammalian adaptation in H5N1 viruses [42]. Phylogenetic analysis of the sequence data indicated that all of the internal gene segments (PB1, PB2, PA, NP, M, and NS) are derived from H9N2 viruses. H7N9 viruses responsible for human cases in 2013, as well as H5N1 viruses that were isolated in humans in 1997, had internal genes from H9N2 viruses [43,44], suggesting that this gene cassette may be a genetic platform for avian strains to cross the species barrier to humans [41]. The HA gene of JX346 belongs to the H10 Eurasian lineage, whereas the NA gene belongs to a sub-cade of the North American lineage. This would suggest that JX346 originated via a reassortment of H9N2 strains circulating in poultry, with H10 viruses contributing the HA and NA genes (Figure 1). This is particularly concerning as this internal six gene cassette is especially successful in poultry and can easily recombine with HA and NA genes that would confer efficient transmission of novel avian influenza strains to humans [43,44]. Somewhat reassuringly, the sequence data indicates JX346 is not fully adapted to humans, the HA protein receptor binding site displays the signature Q-S-G residues at positions 226–228 (H3 numbering) and E190, which are indicative of an avian-like HA receptor binding site [45]. However, the genome of the virus does display some of the genetic hallmarks for mammalian adaptation and virulence; in the HA (A135T, S138A [H3 numbering]), M1 (N30D, T215A), and PB2 (E627K) protein. The polymerase enzyme PB2 displayed E627K, which is often in seen in H7N9 and H5N1 human isolates, suggesting that this mutation was selected for in the infected host as the virus attempted to replicate. Nevertheless, this mutation is insufficient to grant the virus pandemic potential. The JX346 NA was sensitive to the NA inhibitors oseltamivir and zanamivir. When the JX346 virus was grown in embryonated chicken eggs, no mutations were identified in the viral genome, suggesting that the virus is highly adapted to an avian host [46]. Whereas, culture of the virus in MDCK cells produced eight point mutation across the viral genome. Substitutions detected include a complete E627K substitution at the heterogeneous site in PB2 protein, this contrasts with the mixture of E and K observed at this position in the clinical sample. The E627K PB2 substitution has been linked to improved transmissibility and pathogenicity of avian influenza viruses to mammals [46,47,48,49]. In spite of the fact that H10 viruses were never found to harbour a multi-basic amino acid cleavage site in their HA, some isolates were found to exhibit a unusually high pathogenicity for poultry [50,51]. The HA cleavage site of JX3463 displays a single basic amino acid, R, which is coincident with low pathogenicity in poultry [52]. 

In 2014, Chinese authorities notified WHO of a 55-year-old woman with an avian H10N8 infection in Jiangxi province. The woman had no prior exposure to similar cases before the onset of her symptoms and most likely contracted the infection after visiting a local live poultry market, which are a common source of avian influenza dissemination [53]. Two additional cases, a 55-year-old woman and a 75-year-old man were admitted to hospital in the same province in January 2014 [54]. Severe pneumonia and subsequently acute respiratory distress syndrome developed in all three patients, of whom two succumbed 5–6 days after admission [54]. These early human cases of H10N8 and H10N7 infection are significant events as they may represent a virus on the verge of gaining momentum towards pandemic potential.

In an experimental setting, human infection with a H10N7 strain A/turkey/Minnesota/3/79 was demonstrated in 15 healthy human volunteers [55]. The subjects shed the virus and displayed moderate clinical symptoms, and made a full recovery [55]. Moreover, none of the volunteers produced any detectable antibody response, presumably as the multiplication of the virus was insufficient to produce a primary immune response. Experimental infection of mink with H10N4 and H10N7 influenza viruses via intranasal inoculation produced a much more severe infection with interstitial pneumonia and pulmonary lesions, confirming that H10 viruses can be highly pathogenic in certain mammals [56,57]. 

## 4. Receptor Binding Characteristics of H10 HA 

The influenza HA binds to sialylglycoproteins and sialylglycolipids on the surface of host cells [58,59,60]. These sialyl glycans, usually linked to galactose (Gal) in either α2,6 or α2,3 configurations, are the receptors for the viral HA, the binding to which promotes viral attachment, membrane fusion and internalisation of the virus [58,59,60,61,62,63,64,65,66,67]. In the case of the influenza A viruses, which circulate across a number of different mammalian species, the host glycan distribution and binding specificity of the viral HA are significant factors that determine the host range of the virus [58,59,60,61,62,63,64,65,68,69,70]. It is well accepted that the HA of avian influenza A viruses usually display a preference for α 2-3 linked sialyl glycans, whereas the HA of human influenza A viruses preferentially bind α 2-6 linked sialyl glycans [4,58,59,60,61,62,63,64,71,72]. The fact that avian viruses are not capable of efficiently infecting humans without prior adaption is generally attributed to the paucity of α 2-3 linked sialyl glycans along the human upper respiratory tract [4]. Therefore, emerging avian influenza viruses with increased binding to α 2-6 linked sialyl glycans and reduced binding to α 2-3 linked sialyl glycans could pose a major pandemic threat [54]. Swine viruses have been reported to bind both α2,3- and α2,6-SA, but show a greater preference for the latter [58,59,61,62,63,64,71,72]. For effective human-to-human droplet transmission, the viral HA must efficiently bind to human cell surface receptors and possess integral proteins that enable it to efficiently replicate in the cells of the human upper respiratory tract. The spread of H10 influenza to the human population has thus far been rather limited, with no convincing evidence for sustained human-to-human transmission. In humans, avian-like sialyl glycan receptors can be found in non-ciliated cuboidal bronchiolar cells, and on alveolar type II cells in the lower respiratory tract, which might explain why direct human-to-human transmission by coughing and sneezing is inefficient, as the latter would necessitate the presence of avian-type receptors in the upper respiratory tract [45,73,74]. Recently it was demonstrated that direct transmission of H5N1 in mammals is conferred by a finite number of mutations within regions of the receptor binding site (RBS) (N154D, Q222R, S223N) of the HA that overlaps with prominent antigenic positions [75]. This finding adds to the concern that avian viruses can efficiently evolve towards a pandemic strain capable of direct human-to-human droplet transmission [76,77]. 

Insights into the receptor binding specificity of the H10 HA largely come from a number of in vitro studies that used recombinant H10 HA protein and various glycan binding assay platforms [78,79,80,81,82,83]. 

The Stevens laboratory at the CDC expressed and purified the HA ectodomains of the human H10N8 virus, JX346 and the avian H10N7 virus A/green-winged teal/Texas/Y171/2006 and explored their receptor binding specificity using printed glycan microarrays and biolayer interferometry [80] (Table 1). The authors’ results indicated both the human and avian H10 HAs preferentially bind to avian receptor analogues (α2-3 linked sialyl glycans) and there was negligible binding to human receptor analogues (α2-6 linked sialyl glycans). Notably, in the biolayer interferometry experiments, both HAs displayed comparable affinity for the α 2-3-linked 3′SLNLN-b analogue, with an apparent KD of 22.4 µM (A/green-winged teal/Texas/Y171/2006 HA) and 26.5 µM (JX346 HA); no binding was observed for the human receptor analogue 6′SLNLN-b.

Wang et al. generated soluble HA protein from JX346 using a baculovirus expression system and then assayed the sialyl glycan selectivity of the HA using a solid-phase platform, surface plasmon resonance (SPR) and tissue staining platforms [82]. The SPR data revealed that the HA of the H10N8 virus JX346 preferentially binds the avian receptor analogue 3′SLNLN with a KD of 0.4 µM, and shows negligible binding (KD > 1 mM) to the human receptor analogue 6′SLNLN. Similarly, their solid-phase binding data revealed the HA preferentially binds to 3′SLNLN, with negligible binding to 6′SLNLN. The authors went further to use the soluble HA to stain paraffinised human trachea and duck intestinal tract sections. It is worth mentioning that lectin staining has demonstrated that the apical surface of the human trachea largely displays α 2-6 sialyl glycans, whereas the apical surface of the duck intestinal tract largely displays α 2-3 sialyl glycans [73,84]. In line with their binding data, the soluble recombinant H10 HA consistently stained the duck small intestine very well, but did not stain the human trachea.

The Wilson laboratory at Scripps examined the binding properties of recombinant HA from the H10N8 virus JX346 to 3′SLNLN and 6′SLNLN using biolayer interferometry and an ELISA-like binding assay [81] (Table 1). Their results with the ELISA platform showed that the H10 HA preferentially binds to the avian analogue 3′SLNLN, but no detectable binding was observed to the human analogue 6′SLNLN, even at high concentrations of 50 mg/mL. The biolayer interferometry data revealed the H10 HA binds 3′SLNLN with an apparent KD1 of 0.86 µM and KD2 of 0.65 µM, similar to their ELISA data, no detectable binding was observed for 6′SLNLN. The authors went further to characterised H10 HA receptor binding on a custom influenza receptor sialyl glycan microarray, which again revealed that H10 HA has a strong preference for avian α2-3 receptor sialyl glycans, with negligible binding to human-like α2-6 linked sialyl glycans. Furthermore, the authors explored the potential for JX346 HA to acquire human receptor specificity by mutation of key residues in the RBS (Q226L/G228S in H2N2/H3N2 and E190D/G225D in H1N1) associated with pandemic viruses. Interestingly, none of these pairs of mutations produced a dramatic switch in receptor specificity of the H10 HA.

Ramos et al. used flow cytometry in tandem with a solid-phase platform with biotinylated glycans conjugated with a polyacrylamide support to analyse the receptor specificity of recombinant hexahistidine-tagged HAs from the H10N8 virus JX346 and the H10N7 virus A/mallard/Interior Alaska/10BM01929/2010 [54]. Both of these H10 HAs displayed a preference for the avian α2-3 linked sialyl glycans, and low avidity for the human α2-6 linked sialyl glycans. The authors suggest that two amino acids in avian and human H10 HA, namely T135 and S186 that are commonly circulating in human influenza viruses, are associated with changes in receptor binding in other avian influenza A virus subtypes. The authors also demonstrated that the recombinant HA of the JX346 virus is capable of binding to human tracheal samples, suggesting that the virus could potentially attach and replicate in the human upper respiratory tract.

The avian A/chicken/Germany “N”/49 (H10N7) was shown to preferentially bind to α-2,3-SA oligosaccharides using an assay system that measures the ability of ganglioside-coated erythrocytes to undergo virus-mediated haemolysis at a low pH as well as agglutination at pH 7.2 [83]. Recombinant H10 HA of A/Shorebird/DE/10/2004 (H10N7) was capable of binding to ciliated cells on ex vivo human tracheal sections [78]. 

The Skehel group employed biolayer interferometry to show that the recombinant avian H10 from A/mallard/Sweden/51/2002 (H10N2), displayed a 150-fold higher affinity for the avian receptor analogue (3′-SLN=3′-sialyl[N-acetyllactoseamine]) over the human receptor analogue (6′-SLN, 6′-sialyl[N-acetyllactoseamine]) [79]. Another interesting observation was that the binding affinity of the avian H10 HA for the human receptor analogue 6′-SLN was comparable to the HA from the H1 (1918 Spanish influenza) and the H3 (1968 Hong Kong influenza) pandemic viruses [62,85]. The authors noted that the avian H10 HA binds to human receptors more tightly than both avian and human H7 HA isolates. Using microscale thermophoresis experiments, the Skehel group also showed that the purified JX34 H10 HA protein displays an almost comparable affinity for the avian (3′-SLN, KD = 1.81 ± 0.39 mM) and human (6′-SLN KD = 1.39 ± 0.32 mM) analogues [79]. 

Overall, the collective receptor binding data for the human infecting H10N8 virus JX34 suggests that its HA possess preferential specificity for avian type α2-3 linked sialyl glycans; and to switch its specificity towards human-type α2-6 linked sialyl glycans it would need to undergo significant mutation in its RBS beyond the commonly seen mutations in pandemic viruses.

## 5. Structure-Recognition Characteristics Revealed by H10 HA-Receptor Co-Crystallographic Complexes

The structure-recognition characteristics subtypes for most influenza A and B virus HAs have been well characterised [58,59,61,62,63,64,65,80,86,87,88,89,90,91,92,93]. In order to evolve from its present form and gain the pandemic potential for increased transmissibility between humans, the H10 HA will need to undergo mutations that bring about an avian to human receptor preference switch [67]. 

**Figure 2:**
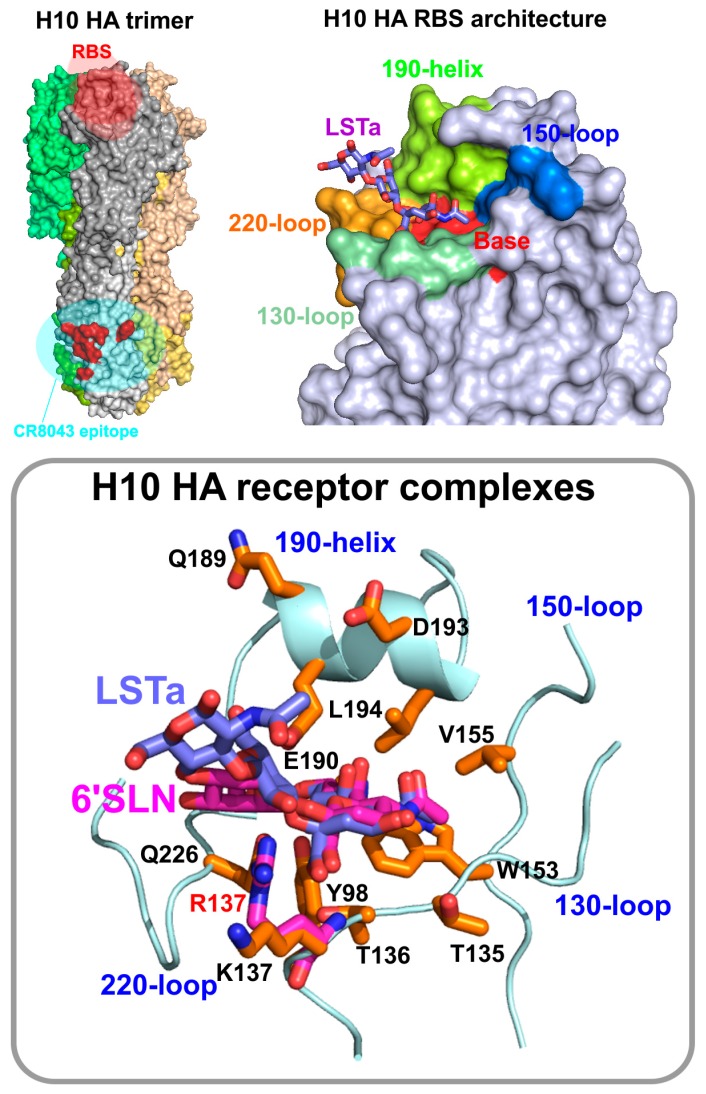
(**Left**) Crystallographic structure of the avian H10 HA trimer (PDB ID: 4CYV) [79]. (**Right**) General architecture of the H10 receptor binding site (RBS). (**Bottom**) The co-crystallographic complex of the avian H10 HA with the avian receptor LSTa and human receptor analogue 6′-SLN [79]. The bound receptor analogues are shown in stick form. The R137 substitution in the JX346 human isolate is shown.

The first reported crystallographic structures of the H10 HAs of the JX34 (H10N8) human isolate and an avian strain A/mallard/Sweden/51/2002 (H10N2) in complex with human and avian receptor analogues were published in *Nature* by the Skehel lab [79]. On a structural level, the H10 HA is not unlike the other HA sub-types [74,94,95]. The quaternary structure of the H10 HA consists of a trimer of identical HA subunits. The HA1 polypeptide encompasses the membrane-distal globular head that harbours the RBS and vestigial esterase domains (Figure 2). The HA2 polypeptide encompasses the stalk region and the CD helix that forms the trimeric coiled-coil. The H10 HA most closely resembles the H7 HA, as both display an elongated 150-loop that penetrates into the RBS and a sharp turn at the amino terminus of α-helix B, which are specific to the H10 and H7 HA clades. Similarly, crystallographic studies of the apo-HAs from A/green-winged teal/Texas/Y171/2006 and JX34by the Stevens laboratory at the CDC and the Wilson laboratory at Scripps, revealed that the H10 HA structures are more structurally related to the HAs (particularly H3 and H7) from its parent group 2 [80,81].

Topographically, the H10 RBS is an indentation on the top of the membrane-distal tip of the influenza HA molecule and is formed of four secondary structural elements, the 130-, 150-, 220-loops and the 190-helix (Figure 2). Five conserved residues, Y98, T136, W153, H183 and P185, form the base of the pocket. Additional residues that form the receptor binding surface include G134, T135, K137 and A138 in the 130-loop; N145, V155 in the 150-loop; G225, Q226, S227, G228 in the 220-loop; and S186, S187, Q189, E190, D193 and L194 in the 190-helix region (Figure 2). 

Pandemic avian influenza viruses display a preference for the human receptor over their native avian receptor. It is likely that the potential avian precursor of the H1 (1918 Spanish influenza) pandemic virus evolved a human receptor binding preference via the E190D mutation in the 190-helix of the RBS. In the case of the avian precursors of the H3N2 1968 Hong Kong and the H2N2 1957 Asian pandemic influenza viruses, the switch to human receptor binding involved a Q226L substitution in the 220-loop [96]. Interestingly, the residues that are known to modulate receptor preference in other HAs, namely positions 186 and 190 in the 190-helix, and residues 223, 225 and 226 in the 220-loop, are conserved in the RBS of the avian H10 HAs and the JX34HA. The structure of the JX34 H10 HA in complex with the human receptor analogue 6′-SLN is very similar to that formed by the avian H10 HA. One notable difference is the contacts between Nag-3 and R137 is the carboxy terminus of the 130-loop in the JX34 HA, whereas in the avian H10 HA this position is substituted with a lysine (K) that does not make any contacts with the receptor. The co-crystal structures of the avian A/mallard/Sweden/51/2002 H10 with the avian receptor analogues sialyllactose-N-tetraose (LSTa) and 3′-SLN is not strikingly different from the complexes seen with HAs of other avian influenza viruses [85,95]. The co-crystallographic complex of the avian A/mallard/Sweden/51/2002 H10 HA with the human receptor analogue 6′-SLN showed that the receptor adopts a cis-configuration about the SA (Sia-1) and galactose-2 (Gal-2) sugars. Notably, this receptor binding configuration is also seen in the HA receptor complexes of the H3N2 1968 Hong Kong and the H2N2 1957 Asian pandemic influenza viruses, that arose via reassortment events between human and avian viruses [85,97]. The H10 HA complex with 6′-SLN displays a 45° difference in the angle of rotation about the Gal-2 C6–C5 bond, which changes the RBS exit trajectory of the human analogue over the 130-loop to a less vertical fashion than that seen in pandemic H1 (1918 Spanish influenza) and H3 (1968 Hong Kong influenza) HA receptor complexes. In contrast, the human receptor analogue exits the side of the H10 HA RBS in a similar trajectory to that seen in the human receptor H7 co-complex. This receptor configuration has been correlated with the presence of L226 in H7 HA RBS, interestingly, in the H10 HA RBS this position is substituted with a Q226 residue. Coincidently, the pandemic H1 (1918 Spanish influenza) HA also displays a Q226 [98]. As a consequence the Gal-2 of the human receptor sits lower (closer to the 130-loop) in the RBS of both the H1 and avian H10 HAs, whereas the avian receptor sits in a higher position (Figure 2). This contrasts with the H2N2 1957 Asian and H3 (1968 Hong Kong influenza) pandemic virus Has, which have a L226 that results in the human receptor sitting higher in the RBS. In addition, the 3-hydroxyl of the Gal-2 sugar is stabilised through contacts with the side chain of Q222 and the main-chain carbonyl at G225 (Figure 2). 

Subsequent to the *Nature* article from the Skehel lab, Wang et al. reported that the structure of the JX34H10 HA bound to the avian (3′SLNLN) and human (6′SLNLN) sialo-pentasaccharide receptor analogues [82]. These authors’ findings show that 3′SLNLN binds in a trans-conformation with three hydrogen bonds between the Sia-1 (main chain carbonyl group of T135) and the 130-loop side-chain hydroxyl group of T136 and the main-chain amino group of residue R137. Two hydrogen bonds are formed with Sia-1 and Q226 in the 220-loop and one hydrogen bond network is mediated by a water between the Sia-1 and Q226 and G228. Two hydrogen bonds are formed with Sia-1 and the residue E190 for the 190-helix. Notably, all of the hydrogen bond interactions between the HA and receptor analogue were converged on the Sia-1 [82]. Interestingly, compared to the 3′SLNLN co-crystal complex, the authors observed only the sialic acid moiety with a poor electron density map in the co-complex with the human analogue 6′SLNLN. Similarly to the avian analogue the 130-loop residues, the 190 helix residue E190 and Q226 in the 220-loop hydrogen bond with the Sia-1 of 6′SLNLN [82]. In the human receptor complex, R137 does not form a hydrogen bond with 6′SLNLN as previously predicted, but instead forms a hydrogen bond with G225 in the 220 loop [82]. A key finding from the study was that residue R137 in the RBS plays a key role for the preferential binding of the HA to the avian receptor analogue. In the apo-HA structure, the side chain of R137 forms a hydrogen bond with the main-chain carbonyl oxygen of G225 in the 220-loop, which is not seen other HA structures. The authors purport that despite the lack of any direct contacts between R137 and 3′SLNLN, this unique hydrogen bridge provides a favourable hydrophilic environment for the exposed hydrophilic glycosidic linkage of the bound 3′SLNLN. Conversely, in the case of 6′SLNLN, the hydrophilic environment formed by the hydrogen bridge would be unfavourable for the binding of the exposed hydrophobic glycosidic linkage of 6′SLNLN. The authors go on to suggest that the differences between their data and those from the aforementioned Skehel group may be due to variations in the experimental setup of the binding assays and the possibility that R137 might be able to adopt different conformations in the RBS. 

Recently, the Wilson laboratory also solved the crystallographic structure of the HA of the H10N8 virus JX34 in complex with avian (3′SLN) and human (6′SLN) receptor analogues [81]. In the 2.85 Å 3′SLN avian receptor complex, the 3′SLN is bound in a trans-conformation with a well-defined electron density was evident for all three sugars with several hydrogen bonds between Sia-1 and the 190-helix, 130-loop (Gal-2 to R137), and 220-loop (Gal-2 to Q226) residues that are highly conserved in avian HA subtypes. Despite its weak affinity for the H10 RBS, the authors managed to soak in the 6′SLN human analogue at 5 mM and resolve the crystal structure of the holo-HA at 3.31 Å resolution. Unlike the avian analogue, the 6′SLN binds in a cis-conformation in a similar fashion to the H7 human receptor complex. The hydrogen bonding pattern seen with the Sia-1 of 3′SLN are also conserved with the Sia-1 of 6′SLN; however, apart from this similarity, the Gal-2 RBS interactions differ markedly. In the 6′SLN complex, the Gal-2 conformation is stabilized by hydrogen bonds with the side chain Q222, S227 main chain amide and the main chain carbonyl of G225.

## 6. Conclusions and Future Perspective 

The pathway for an avian influenza A virus to become a pandemic strain is a complex and multi-factorial process. In recent years, a number of novel avian influenza viruses (H6N1, H7N2, H9N2, and H10N8) have crossed the bird–human species barrier and caused fatal disease in humans. Available data suggest that the currently circulating avian H10 viruses do not appear to be capable of person-to-person droplet transmission as in both the documented Chinese cases the family members and health worker contacts did not contract the infection. Very worryingly, a number of seminal reports have shown that avian strains only require a limited number of gene mutations to achieve droplet transmission and efficient replication in mammals [25,40,42,99,100,101,102]. The avidity of H10 HA for human receptors is low, with low pathogenicity in humans. However, novel subtypes of avian H10 influenza virus exposed to human populations continue to be a major public health concern. It has to be highlighted that a major challenge for future studies is the limited set of synthetic glycans as many human glycans are not represented in current glycan arrays. Continued surveillance is required as part of continuous pandemic preparation activities. We can take some comfort from the knowledge that the H10N8 JX346 human strain was sensitive to NA inhibitors. However, given the extraordinary evolutionary ability of influenza viruses and their propensity for reassortment, there is an imminent possibility that these viruses will improve their transmissibility. We must remain vigilant in our monitoring efforts and vaccine development if we are to defuse this potential ticking pandemic time bomb.

## Figures and Tables

**Table 1:** α2,3 sialyl-glycan specificity of the human A/Jiangxi-Donghu/346/2013 and avian A/green-winged teal/Texas/Y171/2006 H10 HAs.

Glycan Structure	Reference
Neu5Ac(α2-3)Gal(β1-4)Glc (3′SLN)	[79,80,81,82]
Neu5Ac(α2-3)Gal(β1-4)GlcNAc(β1-3)Gal(β1-4)GlcN (3′SLNLN)	[79,80,81,82]
NeuAcα(2-3)-Galβ(1-4)-6-O-sulfo-GlcNAcβ-propyl-NH_2_	[81]
NeuAcα(2-3)-Galβ(1-4)-[Fucα(1-3)]-6-O-sulfo-GlcNAcβ-propyl-NH_2_	[81]
Neu5Acα2-3Galβ1-3[6OSO_3_]GalNAcα	[80]
Neu5Acα2-3Galβ1-4[6OSO_3_]GlcNAcβ	[80]
NeuAcα(2-3)-Galβ(1-3)-6-O-sulfo-GlcNAcβ-propyl-NH_2_	[81]
Neu5Acα2-3Galβ1-3[6OSO_3_]GlcNAc-propyl-NH_2_	[80]
Neu5Acα2-3Galβ1-3(Neu5Acα2-3Galβ1-4)GlcNAcβ	[80]
Neu5Acα2-3Galβ1-3(Neu5Acα2-3Galβ1-4GlcNAcβ1-6)GalNAcα	[80]
Neu5Acα2-3Galβ1-4GlcNAcβ1-2Manα1-3(Neu5Acα2-3Galβ1-4GlcNAc 1-2Manα1-6)Manβ1-4GlcNAcβ1-4GlcNAc β	[80]
Neu5Acα(2-3)-Galβ(1-4)-GlcNAcβ(1-3)-Galβ(1-4)-GlcNAcβ(1-2)-Manα(1-3)-[Neu5Acα(2-3)-Galβ(1-4)-GlcNAcβ(1-3)-Galβ(1-4)-Manα(1-3)-[Neu5Acα(2-3)-Galβ(1-4)-GlcNAcβ(1-3)-Galβ(1-4)-GlcNAcβ(1-2)-Manα(1-6)]-Manβ(1-4)-GlcNAcβ(1-4)-GlcNAcβ	[80]
Neu5Acα2-3Galβ1-3GalNAcα	[80]
Neu5Acα2-3Galβ1-3GlcNAcβ *	[80]
NeuAcα(2-3)-Galβ(1-3)-GalNAcα-Thr-NH_2_	[81]
NeuAcα(2-3)-Galβ(1-3)-[GlcNAcβ(1-6)]-GalNAcα-Thr-NH_2_	[81]
Neu5Acα2-3Galβ1-4GlcNAcβ	[80]
Neu5Acα2-3Galβ1-4GlcNAcβ1-3Galβ1-4GlcNAcβ	[80]
Neu5Acα2-3Galβ1-3GlcNAcβ1-3Galβ1-4GlcNAcβ	[80]
NeuAcα(2-3)-Galβ(1-4)-GlcNAcβ(1-3)-[NeuAcα(2-3)-Galβ(1-4)-GlcNAcβ(1-6)]-GalNAcα-Thr-NH_2_	[81]
Neu5Acα2-3Galβ1-4GlcNAcβ1-3Galβ1-4GlcNAcβ1-3Galβ1-4GlcNAcβ	[80]
NeuAcα(2-3)-Galβ(1-4)-GlcNAcβ(1-3)-Galβ(1-4)-GlcNAcβ(1-3)-[NeuAcα(2-3)-Galβ(1-4)-GlcNAcβ(1-3)-Galβ(1-4)-GlcNAcβ(1-6)]-GalNAcα-Thr-NH_2_	[81]
Neu5Acα2-3Galβ1-4GlcNAcβ1-3Galβ1-3GlcNAcβ	[80]
NeuAcα(2-3)-Galβ(1-4)-GlcNAcβ(1-2)-Manα(1-3)-[NeuAcα(2-3)-Galβ(1-4)-GlcNAcβ(1-2)-Manα(1-6)]-Manβ(1-4)-GlcNAcβ(1-4)-GlcNAcβ-Asn-NH_2_	[81]
Neu5Acα2-3Galβ1-3GalNAcα	[80]
NeuAcα(2-3)-Galβ(1-4)-GlcNAcβ(1-3)-Galβ(1-4)-GlcNAcβ(1-2)-Manα(1-3)-[NeuAcα(2-3)-Galβ(1-4)-GlcNAcβ(1-3)-Galβ(1-4)-GlcNAcβ(1-2)-Manα(1-6)]-Manβ(1-4)-GlcNAcβ(1-4)-GlcNAcβ-Asn-NH_2_	[81]
Neu5Acα2-3Galβ1-4(Fucα1-3)GlcNAcβ1-3Galβ1-4(Fucα1-3)GlcNAcβ1-3Ga β1-4(Fucα1-3)GlcNAcβ *	[80]

* Low binding observed to the A/Jiangxi-Donghu/346/2013 H10 HA.
